# The Significance of Prolonged and Saddleback Fever in Hospitalised Adult Dengue

**DOI:** 10.1371/journal.pone.0167025

**Published:** 2016-12-09

**Authors:** Deborah HL Ng, Joshua GX Wong, Tun-Linn Thein, Yee-Sin Leo, David C. Lye

**Affiliations:** 1 Communicable Disease Centre, Institute of Infectious Diseases and Epidemiology, Singapore, Singapore; 2 Yong Loo Lin School of Medicine, National University of Singapore, Singapore, Singapore; 3 Lee Kong Chian School of Medicine, Nanyang Technological University, Singapore, Singapore; 4 Saw Swee Hock School of Public Health, Singapore, Singapore; Institute of Tropical Medicine (NEKKEN), Nagasaki University, JAPAN

## Abstract

Dengue fever is gaining importance in Singapore with an increase in the number of cases and mortality in recent years. Although prolonged and saddleback fever have been reported in dengue fever, there are no specific studies on their significance in dengue. This study aims to examine the prevalence of prolonged and saddleback fever in dengue as well as their associations with dengue severity. A total of 2843 polymerase-chain reaction (PCR) confirmed dengue patients admitted to Tan Tock Seng Hospital from 2004 to 2008 were included in the study. Sixty-nine percent of them were male with a median age of 34 years. Prolonged fever (fever > 7 days duration) was present in 572 (20.1%) of patients. Dengue hemorrhagic fever (DHF), dengue shock syndrome (DSS) and severe dengue (SD) were significantly more likely to occur in patients with prolonged fever. Mucosal bleeding, anorexia, diarrhea, abdominal pain, nausea or vomiting, lethargy, rash, clinical fluid accumulation, hepatomegaly, nosocomial infection, leukopenia, higher neutrophil count, higher hematocrit, higher alanine transaminase (ALT) and aspartate transaminase (AST), higher creatinine, lower protein and prolonged activated partial thromboplastin time (APTT) were significantly associated with prolonged fever but not platelet count or prothrombin time (PT). Saddleback fever was present in 165 (5.8%). Although DHF and SD were more likely to occur in patients in those with saddleback fever, DSS was not. Compared with prolonged fever, saddleback fever did not show many significant associations except for diarrhea, abdominal pain, clinical fluid accumulation, hematocrit and platelet change, and lower systolic blood pressure. This study demonstrates that prolonged fever may be associated with various warning signs and more severe forms of dengue (SD, DSS, DHF), while saddleback fever showed associations with DHF and SD but not DSS. The presence of prolonged or saddleback fever in dengue patients should therefore prompt detailed evaluation for complications of dengue, as well as early investigation to evaluate for development of nosocomial infection.

## Background

Dengue infection is an acute mosquito-borne viral infection of which there are four serotypes, namely DENV-1, DENV-2, DENV-3, and DENV-4, transmitted by the *Aedes* mosquito. Dengue is now endemic in over 100 countries with as much of 50% of the world’s population at risk of the disease. [1] Global estimates suggest that there may be as many as 390 million dengue infections a year, with about 96 million cases manifesting clinically. [2]

Dengue fever has become an infection of public health importance in Singapore since 2004, when a record of 9459 cases was notified that year. [3] In 2013, a total of more than 22,000 cases of dengue were notified with a total of 8 dengue deaths reported. A study conducted among hospitalized adult dengue patients in Singapore reported that the incidence of dengue hemorrhagic fever (DHF) was 21.7% and dengue shock syndrome (DSS) 3.4%. [4]

With this rapidly increasing incidence of dengue cases worldwide, the World Health Organization has recommended that research efforts be directed towards reducing the morbidity and mortality associated with DHF. [5] A meta-analysis found that factors such as nausea or vomiting, abdominal pain, gastrointestinal bleeding, hemoconcentration, plasma leakage, elevated transaminases, thrombocytopenia and prolonged coagulation were significantly associated with the development of DSS. [6] The presence of such factors has been useful in alerting clinicians to the development of complications such as DSS or DHF.

Prolonged fever has been reported in dengue infection, with some studies reporting fever duration lasting as much as up to 12 days. [7] The phenomenon of saddleback fever, with a biphasic pattern, has also been reported in dengue. [8,9] Although these reports suggest that saddleback fever may aid in the clinical diagnosis of dengue, the prognostic significance of both prolonged and saddleback fever have not been well studied. The aim of this study was to examine the prevalence of prolonged and saddleback fever as well as their associations with dengue severity.

## Methods

### Data collection

All patients hospitalized in Tan Tock Seng Hospital, Singapore from 1 January 2004 to 31 December 2008 with acute dengue infection were included in this retrospective cohort study. Confirmed dengue cases were defined as those with a positive polymerase chain reaction (PCR) described by Barkham at el. [10] The study aimed to recruit patients who were in the early phase of dengue infection; hence patients who were serology positive but PCR negative were excluded from the study. Objective information on dates of fever starting and ending was unavailable in such cases.

Data on patients’ demographic information, co-morbid conditions, symptoms, signs and laboratory results were collected from chart review including electronic medical records. During the study period, a standardized dengue care path was in use to manage all hospitalized dengue patients, ensuring consistency of documentation of key clinical information and ordering of essential laboratory tests. All collected data were identified from date of admission to date of discharge.

### Definitions

Fever was defined as a tympanic temperature >37.5°C and duration was calculated from the date of onset, as reported by the patient, to the date of final defervescence, as measured during the hospital admission. Prolonged fever was defined as fever lasting more than 7 days. Saddleback fever refers to biphasic fever with an initial peak that remits and appears again. In this study, saddleback fever was defined as temperature >37.5°C with defervescence of at least one day, followed by a second peak lasting at least one day. Patients without prolonged or saddleback fever were considered part of the control group. The assessment of dengue warning signs, DHF, DSS and severe dengue (SD) were made in patients with laboratory confirmed dengue at the time of hospital discharge using available information from hospital admission to discharge, as defined by the World Health Organization dengue guidelines in 1997 and 2009 respectively. [11,12] Dengue hemorrhagic fever, according to the 1997 dengue guideline, was defined as the presence of fever, or history of acute fever, lasting two to seven days, hemorrhagic tendencies, thrombocytopenia of 100 cells per mm^3^ or less and evidence of plasma leakage due to increased vascular permeability. Dengue shock syndrome was defined as the presence of circulatory failure in addition to the four criteria for DHF.

According to the 2009 guideline, severe dengue is defined as the presence of severe plasma leakage, severe bleeding and severe organ involvement. Plasma leakage was defined as a change in hematocrit of 20% of more from baseline or clinical signs such as pleural effusion or ascites.

Clinical fluid accumulation was defined as the presence of lung dullness or shifting dullness on examination or radiological evidence of pleural effusion or ascites. Hepatomegaly was defined as the presence of a palpable liver below the costal margin or radiological evidence of hepatomegaly. Mucosal bleeding refers to bleeding occurring from the gums or nose, hemoptysis, hematemesis, melena, bleeding per rectum or menorrhagia. Warning signs include: abdominal pain or tenderness, persistent vomiting for 2 days or more, clinical fluid accumulation, mucosal bleeding, lethargy, hepatomegaly or hematocrit change of more than 20% concurrent with thrombocytopenia of less than 50 x 10^9^/L on the same day. A standardized dengue care path was used to manage patients. Treatment decision was made by individual doctors. Patients without clinical bleeding may be given prophylactic platelet transfusion if their platelet count fell below 20 x 10^9^/L or if there was evidence of clinical bleeding, regardless of severity during the study period. Clinically significant bleeding was defined as any bleeding from the gastrointestinal tract or bleeding requiring blood product transfusion. Nosocomial infection was defined as infection acquired after 48 hours of hospital admission. Urinary tract infection (UTI) was defined as a positive urine culture with clinical features of UTI. Bloodstream infection was defined as a clinically significant bacterium isolated from blood culture. Pneumonia was defined by the presence of new consolidation on chest X-ray with compatible clinical signs and symptoms. *Clostridium difficile* infection was defined as positive stool *Clostridium difficile* toxin with diarrhea.

### Statistical methods

Statistical analysis was performed using R3.0.2. Chi-square and Fisher’s exact test were used to test for associations in categorical variables and Mann-Whitney U test was carried out to test for differences in continuous variables. Multinomial regression was performed to identify clinical and laboratory parameters that were independently different between patients with prolonged and saddleback fever, with the control group as reference. Variables were removed if they were statistically insignificant on both outcomes. The model was adjusted for potential confounders (age and gender). All tests were performed at a 5% significance level.

### Ethics statement

The study was approved by the Domain Specific Review Board (DSRB) of the National Healthcare Group.

All patient data collected were anonymized.

## Results

### Cohort description

There were a total of 2874 adult patients with PCR-positive dengue, of which 31 were excluded as they fulfilled overlapping criteria for prolonged and saddleback fever. The median age was 34 years of age (5^th^- 95^th^ percentile: 17–59) and males comprised 1971 (69.3%) of study subjects. Medical co-morbidities were documented in 406 (14.1%) of patients. The median duration of fever at the time of presentation was 4 days (5^th^– 95^th^ percentile: 2–6).

In terms of dengue severity at the point of discharge, 1865 (65.6%) patients had dengue with warning signs, 576 (20.3%) had DHF and 352 (12.4%) SD. Among the patients with SD, there were 77 patients with plasma leakage, 165 experienced severe bleeding and 72 with severe organ impairment. The remaining 38 experienced more than 1 outcome of plasma leakage, severe bleeding or severe organ impairment. Eighty-one patients had dengue with no warning signs, of which most did not experience prolonged or saddleback fever (n = 67).

The median length of hospital stay was 5 days (5th- 95th percentile: 3–8). Adverse outcome was uncommon, with 13 (0.4%) patients requiring admission to the intensive care unit and 2 deaths reported. Neither of the deaths had prolonged fever, although one had saddleback fever. The median duration of fever from onset to first defervescence was 6 days (5th- 95th percentile: 4–9).

### Associations of prolonged and saddleback fever

Prolonged fever was present in 572 (20.1%) patients, of whom 40 (7%) had fever of more than 10 days and 6 (1%) more than 14 days. The following factors (Table 1) during hospitalization were associated with prolonged fever: mucosal bleeding (p<0.01), anorexia (p = 0.01), abdominal pain(p<0.01), nausea or vomiting (p<0.01), lethargy(p<0.01), rash (p = 0.01), clinical fluid accumulation (p = 0.02), hepatomegaly (p<0.01), leukopenia (p<0.01), higher neutrophil count (p<0.01), higher hematocrit (p = 0.04), higher alanine transferase (ALT)(p<0.01) and aspartate transferase (AST) (p<0.01), higher creatinine (p<0.01), lower protein (p<0.01) and prolonged activated partial thromboplastin time (APTT) (p<0.01) but not platelet count (p = 0.17) or prothrombin time (PT) (p = 0.7).

**Table 1:** Demographic, clinical and laboratory features of prolonged and saddleback fever in adult dengue.

Demographics	Control (n = 2106)	Prolonged fever (n = 572)	p-value^#^	Saddleback fever (n = 165)	p-value^#^
Male	1494(70.9)	364(63.6)	<0.01	113(68.5)	0.56
Age (years)	34(16–57)	34(16–58)	0.49	34(18–60)	0.27
Diabetes mellitus	69(3.3)	17(3)	0.79	3(1.8)	0.48
Hypertension	174(8.3)	30(5.2)	0.02	14(8.5)	0.88
Cardiac	25(1.2)	9(1.6)	0.53	3(1.8)	0.45
**Symptoms**					
Headache	1075(51)	311(54.4)	0.16	87(52.7)	0.17
Eye pain	41(1.9)	9(1.6)	0.73	3(1.8)	0.99
Myalgia/arthralgia	1542(73.2)	441(77.1)	0.07	128(77.6)	0.22
Gum bleed	394(18.7)	156(27.3)	<0.01	35(21.2)	0.41
Nose bleed	81(3.8)	35(6.1)	0.02	3(1.8)	0.28
Menorrhagia	74(3.5)	28(4.9)	0.13	5(3)	0.99
Anorexia	1268(60.2)	378(66.1)	0.01	114(69.1)	0.02
Diarrhea	687(32.6)	245(42.8)	<0.01	69(41.8)	0.02
Abdominal pain	674(32)	235(41.1)	<0.01	62(37.6)	0.14
Rash	1228(58.3)	368(64.3)	0.01	97(58.8)	0.9
Nausea/vomiting	1514(71.9)	446(78)	<0.01	125(75.8)	0.28
**Warning signs**					
Clinical fluid accumulation	63(3)	29(5.1)	0.02	15(9.1)	<0.01
Mucosal bleeding	546(25.9)	208(36.4)	<0.01	50(30.3)	0.22
Lethargy	504(23.9)	176(30.8)	<0.01	47(28.5)	0.19
Hepatomegaly	32(1.5)	22(3.8)	<0.01	3(1.8)	0.74
Hematocrit and platelet change	254(12.1)	103(18)	<0.01	41(24.8)	<0.01
**Vital signs**					
Systolic blood pressure, mmHg	98 (85–115)	95 (80–100)	<0.01	95 (81–110)	<0.01
Temperature,°C	38.6 (37.3–39.9)	39 (37.8–40)	<0.01	38.6 (37.8–39.9)	0.24
Pulse, per minute	94 (75–116)	95 (80–116)	<0.01	95 (75–118)	0.42
**Laboratory findings**					
White cell count,10^9^/L	2 (1.1–4.3)	1.8 (0.9–3.9)	<0.01	2 (1–4)	0.1
Neutrophil,10^9^/L	32 (16–51)	34 (16–53)	<0.01	33 (20.2–51.3)	0.17
Hematocrit, %	45.2 (38–51)	45 (38–51)	0.04	45.4 (37.9–53)	0.14
Platelet,10^9^/L	35 (9–89)	33 (10–81)	0.17	26 (7–66)	<0.01
Aspartate aminotransferase, U/L	99 (31–513)	135 (34–943)	<0.01	114 (33–595)	0.08
Alanine aminotransferase, U/L	57 (18–386)	76 (18–595)	<0.01	62 (19–457)	0.37
Creatinine, umol/L	81 (52–114)	77 (50–110)	<0.01	83 (53–118)	0.35
Total protein, g/L	66 (55–76)	65 (53–76)	<0.01	65.6 (54–75)	0.06
PT, seconds	13.5 (12.2–15.7)	13.5 (12.2–15.7)	0.7	13.7 (12.7–16.2)	<0.01
PTT, seconds	41.2 (32.6–54.4)	43.1 (33.1–58.6)	<0.01	42.5 (33.6)	<0.01

^#^p values are compared against control group

Saddleback fever was present in 165 (5.8%) patients. Compared with prolonged fever, saddleback fever did not show many significant associations with the majority of clinical and laboratory variables except for anorexia (p = 0.02), diarrhea (p = 0.02), clinical fluid accumulation (p<0.01), hematocrit and platelet change (<0.01), thrombocytopenia (p< 0.01) and prolonged PT (p<0.01) and APTT (p<0.01).

### Multivariate analysis

Patients with gum bleeding, diarrhea, abdominal pain, hepatomegaly, higher neutrophil count and AST were statistically more likely to experience prolonged fever. Clinical fluid accumulation, hematocrit and platelet change, and lower platelet counts were associated with saddleback fever. Both prolonged and saddleback fever had statistically lower white cell count compared with controls. (Table 2)

**Table 2:** Multivariate analysis of associations of prolonged and saddleback fever, adjusted for age and gender.

	Adjusted odds ratio and 95% confidence interval				
	Control	Prolonged fever	p-value	Saddleback fever	p-value
Gum bleeding	Reference	1.5 (1.2–1.8)	<0.01	1.02 (0.7–1.5)	0.92
Diarrhea		1.4 (1.2–1.7)	<0.01	1.4 (0.98–1.9)	0.07
Abdominal pain		1.3 (1.03–1.5)	0.02	1.03 (0.7–1.5)	0.87
Clinical fluid accumulation		1.2 (0.7–2)	0.5	2.1 (1.1–3.9)	0.03
Hepatomegaly		2 (1.1–3.5)	0.02	0.9 (0.3–3)	0.84
Hematocrit and platelet change		1.3 (0.99–1.8)	0.05	1.7 (1.1–2.6)	0.02
White cell count, 10^9^/L		0.8 (0.7–0.9)	<0.01	0.8 (0.7–0.99)	0.04
Neutrophils,10^9^/L		1.02 (1.01–1.03)	<0.01	1.01 (0.99–1.03)	0.13
Platelet, 10^9^/L		0.998 (0.994–1.002)	0.50	0.987 (0.979–0.995)	<0.01
AST, U/L		1.0004 (1.00009–1.0007)	0.01	0.99 (0.99–1.00)	0.58

### Outcomes and complications of dengue infection in hospitalised adult patients with prolonged and saddleback fever

Dengue hemorrhagic fever was significantly more likely to occur in those with prolonged fever, compared with the control group (25.7% versus 18.3%, p<0.01). Prolonged fever was also more prevalent in DSS (5.4% versus 3.1%, p <0.01) and SD (18.2% versus 10.4%, p<0.01), compared with controls. Similarly, DHF and SD were significantly more likely to occur in patients with saddleback fever, compared with those who did not. (Table 3)

**Table 3:** Outcomes and complications occurring in hospitalized adult dengue patients.

	Control (n = 2106)	Prolonged fever (n = 572)	p-value^#^	Saddleback fever (n = 165)	p-value^#^
Dengue with warning signs (2009)	1314(62.4)	433(75.7)	<0.01	117(70.9)	0.03
Dengue hemorrhagic fever	385(18.3)	147(25.7)	<0.01	44(26.7)	0.01
Dengue shock syndrome	65(3.1)	31(5.4)	<0.01	9(5.5)	0.1
Severe dengue	219(10.4)	104(18.2)	<0.01	29(17.6)	<0.01
Severe plasma leakage	63(3)	29(5.1)	0.020	15(9.1)	<0.01
Severe bleeding	130(6.2)	53(9.3)	0.010	13(7.9)	0.4
Severe organ involvement	49(2.3)	38(6.6)	<0.01	5(3)	0.6
Nosocomial infections	(0)				
Urinary tract infection	2(0.1)	7(1.2)	<0.01	2(1.2)	0.04
Pneumonia	7(0.3)	8(1.4)	0.01	1(0.6)	0.49
*Clostridium difficile*	0(0)	0(0)	NA	0(0)	NA
Bacteremia	1(0)	6(1)	<0.01	0(0)	NA
Intensive care unit admission	9(0.4)	2(0.3)	0.570	2(1.2)	0.19
Length of stay (days)	5(3–7)	6(4–9)	<0.01	6(4–8)	<0.01
Death	1(0)	0(0)	NA	1(0.6)	NA

^#^p values are compared against control group

Dichotomous variables are expressed as number and percentage in parentheses and continuous variables as median and interquartile ranges.

Patients with prolonged fever were more likely to have fluid (crystalloid) infusion compared with the control group (89.3% versus 84.3%, p<0.01) but not platelet transfusion (17.7% versus 18.4%, p = 0.67). Patients with saddleback fever were not more likely to have fluid (crystalloid) infusion compared with the control group (88.5% versus 84.3%, p = 0.15). However, patients with saddleback fever were more likely to receive platelet transfusion compared with the control group (30.3% versus 18.4%, p<0.01). Two patients in the prolonged fever group (0.35%), two patients in the saddleback fever group (16.7%) and 8 patients in the control group (0.38%) required packed cell transfusions but these differences did not reach statistical significance.

Two patients with prolonged fever, two with saddleback fever and 9 patients in the control group required admission to the intensive care unit (ICU). There was no significant association between ICU admission and prolonged fever. The duration of hospital stay was significantly longer for patients with prolonged fever (6 [5^th^– 95th percentile: 4–9] versus 5 [5^th^– 95th percentile: 3–7] days) and saddleback fever (6 [5^th^– 95^th^ percentile: 4–8] versus 5 [5^th^– 95^th^ percentile: 3–7] days), compared with those who did not.

Among patients who developed nosocomial infection, those with prolonged fever were significantly more likely to develop bacteremia, pneumonia and UTI than those who did not. None of the patients in either group developed *Clostridium difficile* infection. There were a total of two deaths, one of whom died from DHF and the second from septic shock secondary to pneumonia.

## Discussion

This study documented that prolonged fever was not an uncommon feature of adult dengue fever, with one fifth of patients experiencing fever more than 7 days, and may be associated with increased incidence of complications of dengue severity such as DHF, DSS or SD. Similarly, findings of a study conducted in India among children with dengue fever found that prolonged fever of more than 7 days was associated with the development of DHF and DSS. [13] Although saddleback fever was not as frequently seen (5.8%) in this cohort, this study demonstrated that it was not rare in adult dengue.

We also found that selected symptoms and warning signs were more prevalent in the prolonged fever group than the control and saddleback fever groups (Table 1). Findings of the multivariate analysis supported these observations which showed that gum bleeding, diarrhea and abdominal pain were independently associated with prolonged fever, even after adjusting for covariates.

Clinical fluid accumulation was more likely to occur in saddleback fever (aOR 2.1 [95% confidence interval, 1.1–3.9]) compared with the control group, as was receipt of platelet transfusion. Our study also demonstrated that saddleback fever was more likely to be associated with thrombocytopenia compared to the control group, which in turn could explain the observation that they were also more likely to receive platelet transfusion. A study among pediatric patients with dengue shock syndrome found that those who received platelet transfusions were significantly more likely to develop pulmonary edema. [14] Similarly, another study among adult dengue patients receiving prophylactic platelet transfusion also found that they received significantly more amounts of fluid volume compared with those who did not. [15] This may suggest the reason for the association between clinical fluid accumulation and saddleback fever. Saddleback fever may also have occurred as a response to the platelet transfusion, although we did not collect data to support this hypothesis.

In addition, a study conducted in Taiwan among patients with DHF/DSS found that patients with prolonged fever (>5 days) were at high risk for concurrent bacteremia. [16] This correlated with the findings of our study, which found that prolonged fever was significantly associated with nosocomial infection. Analysis of adult dengue deaths that occurred in Singapore between 2004 and 2008 found that bacteremia was documented in 14% of patients. [17] Another similar study of 9 deaths among adult dengue patients found that secondary bacteremia was a contributing factor in four patients. [18] Although duration of fever was not recorded among these patients, prolonged fever should alert clinicians about the increased risk of mortality and to look out for other serious concurrent infection, which may indicate the need for empiric antimicrobial therapy.

Duration of dengue viremia has been demonstrated to correlate with duration of fever, meaning that viremia persisted while the patient was febrile and subsided as the fever subsided. [19] It may be postulated that prolonged fever may equate to prolonged dengue viremia, although there is no current study to prove this theory. In fact, a study among children with dengue infection found that there was no correlation between disease severity and duration of dengue viremia. [20] A study of adult patients with dengue infection reported that although patients suffering from fatal DHF at the time of death had significant viremia, there was no correlation between the level of dengue viremia and disease severity. [21]

Elevated ALT and AST are well-known to occur in dengue. In particular, a study comparing liver aminotransferase values found that maximum values were more likely to occur during febrile and critical phases but not during the convalescent phase. [22] As such, clinicians should be aware of the possibility of more severe liver injury in patients with prolonged fever, as we demonstrated in this study.

One of the strengths of this study is the large sample size with data collected over a period of time, with a well-characterized population. Furthermore, only laboratory-confirmed cases of dengue were included in the study. This meant that the natural progression of the fever in dengue could be more accurately charted. One limitation of the study is that onset of fever relied on self-reporting by patients. Over- or under-reporting of the onset of fever prior to admission could in turn affect the number of patients found to have prolonged fever. Patients admitted to hospital were also likely to be suffering from more severe forms of dengue introducing selection bias. Another limitation is that our study was conducted at a single center and comprised only adult patients. This suggests that the study results may not be generalized to children.

This is the first known study that has specifically reported on the significance of prolonged and saddleback fever in adult dengue infection. Findings of this study have demonstrated that prolonged fever may be associated with various warning signs and more severe forms of dengue (DHF, DSS, SD), while saddleback fever showed associations with DHF and SD but not DSS. The presence of prolonged or saddleback fever in dengue patients should therefore prompt detailed evaluation for complications of dengue, as well as early investigation to evaluate for development of nosocomial infection.
